# Human mesenchymal stromal cells release functional mitochondria in extracellular vesicles

**DOI:** 10.3389/fbioe.2022.870193

**Published:** 2022-08-19

**Authors:** Matthew A. Thomas, Megan J. Fahey, Brenna R. Pugliese, Rebecca M. Irwin, Marc A. Antonyak, Michelle L. Delco

**Affiliations:** ^1^ Cornell University College of Veterinary Medicine, Department of Clinical Sciences, Ithaca, NY, United States; ^2^ Cornell University College of Veterinary Medicine, Department of Molecular Medicine, Ithaca, NY, United States

**Keywords:** MSCs, mitoEVs, secretome, mitochondria, mitochondrial transfer, regenerative orthobiologic

## Abstract

Cartilage and other skeletal soft tissues heal poorly after injury, in part due to their lack of vascularity and low metabolic rate. No pharmacologic approaches have proven effective in preventing chronic degenerative disease after joint injury. Mesenchymal stromal cells (MSCs) have been investigated for their ability to treat pain associated with osteoarthritis (OA) and preserve articular cartilage. Limitations of MSCs include variability in cell phenotype, low engraftment and retention rates, and inconsistent clinical outcomes. Therefore, acellular biologic therapies such as extracellular vesicles (EVs) are currently being investigated. MSC-derived EVs have been found to replicate many of the therapeutic effects of their cells of origin, but the mechanisms driving this remain unclear. Recent evidence in non-orthopedic tissues suggests MSCs can rescue injured cells by donating mitochondria, restoring mitochondrial function in recipient cells, preserving cell viability, and promoting tissue repair. Our group hypothesized that MSCs package mitochondria for export into EVs, and that these so-called “mitoEVs” could provide a delivery strategy for cell-free mitochondria-targeted therapy. Therefore, the goals of this study were to: 1) characterize the vesicle fractions of the MSCs secretome with respect to mitochondrial cargoes, 2) determine if MSC-EVs contain functional mitochondria, and 3) determine if chondrocytes can take up MSC-derived mitoEVs. We isolated exosome, microvesicle, and vesicle-free fractions from MSC-conditioned media. Using a combination of dynamic light scattering and nanoparticle tracking, we determined that MSC-EV populations fall within the three size categories typically used to classify EVs (exosomes, microvesicles, apoptotic bodies). Fluorescent nanoparticle tracking, immunoblotting, and flow cytometry revealed that mitochondrial cargoes are abundant across all EV size populations, and mitoEVs are nearly ubiquitous among the largest EVs. Polarization staining indicated a subset of mitoEVs contain functional mitochondria. Finally, flow cytometry and fluorescent imaging confirmed uptake of mitoEVs by chondrocytes undergoing rotenone/antimycin-induced mitochondrial dysfunction. These data indicate that MSCs package intact, functional mitochondria into EVs, which can be transferred to chondrocytes in the absence of direct cell-cell interactions. This work suggests intercellular transfer of healthy MT to chondrocytes could represent a new, acellular approach to augment mitochondrial content and function in poorly-healing avascular skeletal soft tissues.

## 1 Introduction

Cartilage is responsible for dissipating mechanical forces and providing a near frictionless articular surface for locomotion (Sophia Fox et al., 2009). Injury to the articular surface leads to chondrocyte death and cartilage matrix degradation. This cellular and structural damage initiates cycles of inflammation and irreversible tissue degeneration, culminating in end-stage osteoarthritis (OA) (Madry et al., 2011), a leading cause of chronic pain and disability worldwide (Lawrence et al., 2008). Cartilage and other skeletal soft tissues such as meniscus, ligament and intervertebral disc have a low metabolic rate, poor vascularization and as such, possess limited healing capacity (Bianchi and Sinigaglia, 2012). Further, no current therapeutics can slow progression of OA after joint injury (Sarzi-Puttini et al., 2005).

Recent studies have shown that mitochondrial dysfunction is one of the earliest responses of chondrocytes to mechanical injury, implicating mitochondria (MT) as an important mediator of injury-induced OA (Blanco et al., 2011; Goetz et al., 2017; Delco et al., 2018a; Bonnevie et al., 2018; Liu et al., 2019). We have found that the mitoprotective peptide SS-31 can improve MT function, preserve chondrocyte viability, and prevent tissue loss following cartilage injury (Delco et al., 2018b; Bartell et al., 2020). These findings support the concept of protecting MT function after acute injury to prevent cartilage loss, however MT-targeted strategies to improve healing of degenerated articular cartilage have not been investigated.

Tissue healing is an energy intensive process, and MT dysfunction mediates many degenerative diseases, including Parkinson’s (Winklhofer and Haass, 2010), cardiomyopathy (Pacak et al., 2015), and pulmonary fibrosis (Watson et al., 2016). MT dysfunction has been less well studied in cartilage, in part because chondrocytes are largely glycolytic and do not heavily rely on MT for energy production during homeostasis (Bianchi and Sinigaglia, 2012). However, MT also act as signaling hubs in cellular processes such as apoptosis and autophagy, and are critical organelles for maintenance of cartilage homeostasis (Loeser, 2011). Inflammation that occurs in OA is associated with a shift towards glycolysis for energy production in chondrocytes (Zheng et al., 2021). This metabolic shift may represent an attempt to restore energy capacity after MT dysfunction reduces an already limited supply (Mobasheri et al., 2017). Therefore, replacing dysfunctional MT that have been lost to injury and/or disease may promote healing in avascular, metabolically quiescent tissues such as articular cartilage.

Intercellular MT transfer, a process whereby injured and dysfunctional cells recruit MSCs to donate functional MT, has been shown to restore MT function in energy-expensive tissues such as neurons (Berridge et al., 2016), cardiomyocytes (Pacak et al., 2015) and renal tubular epithelia (Konari et al., 2019), as well as more quiescent, poorly vascularized tissues such as the corneal epithelia (Jiang et al., 2016). *In vivo* murine models of acute lung injury revealed that MSC-mediated MT transfer protects against tissue degeneration after pulmonary injury (Islam et al., 2012; Jackson et al., 2016). Though the mechanisms mediating intercellular MT transfer have not been fully elucidated, evidence from a variety of cell and tissue types implicate processes requiring direct cell-cell contact, including filapodial extensions, tunneling nanotubules, gap junction formation, and cell-cell fusion events (Chinnery et al., 2008; Larsson et al., 2008; Murray and Krasnodembskaya, 2019).

In addition to direct cell-cell MT transfer, our group and others have documented apparent non-contact MT transfer via extracellular vesicles (EVs). EVs are small, membrane bound structures released by all cell types. EVs serve as vehicles for cell-cell signaling and orchestrate many biologic processes including inflammation, immunomodulation, homeostasis, metastasis, and tissue repair (Lai et al., 2010; Luwig and Giebel, 2012; Yuana et al., 2013; Kogure et al., 2020). While EV populations are heterogenous and remain largely uncharacterized, they are most commonly classified into three categories based on size and mode of biogenesis: exosomes, microvesicles (MVs), and apoptotic bodies (Gould and Raposo, 2013). Recent work has identified mitochondria-specific cargoes, including DNA, RNA, and proteins in EVs originating from many cell types, including fibroblasts (Sansone et al., 2017), neurons (Peruzzotti-Jametti et al., 2021), and MSCs (Phinney et al., 2015). Furthermore, intact MT have been identified in larger vesicles derived from adipocytes (Crewe et al., 2021) and MSCs (Phinney et al., 2015; Morrison et al., 2017). In one study, at least 60% of human plasma-derived EVs contained MT, and MT cargoes were ubiquitous in the very largest vesicles (Zhang et al., 2020). Further, EV-mediated MT transfer has been documented between MSCs and professional phagocytic cells; *in vitro,* macrophages were found to take up MSC-derived EVs containing MT and incorporate them into their mitochondrial networks (Phinney et al., 2015; Morrison et al., 2017). This process of stem cell to phage mitoEV-mediated transfer has also been documented from neural stem cells (NSCs) to mononuclear phagocytes, with transfer restoring ATP capacity, upregulating oxidative phosphorylation, and reducing inflammation (Peruzzotti-Jametti et al., 2021). However, MSC-derived mitoEV transfer has not been demonstrated to chondrocytes. Intriguingly, our group has reported MSC-chondrocyte MT transfer in direct coculture and observed MSCs apparently shedding MT into the extracellular space during co-culture with injured chondrocytes (Bennett et al., 2019; Fahey et al., 2022). However, it was unclear if these MT were packaged within EVs (mitoEVs). Therefore, the goals of this study were to characterize EVs produced by murine and human bone marrow derived-MSCs (including mitochondrial cargoes), determine if MSC-mitoEVs contain functional mitochondria, and investigate whether chondrocytes are capable of taking up MSC-derived mitoEVs. While live MSCs have shown promise as an orthobiologic therapy (Kingery et al., 2019), the therapeutic use of live cells presents significant clinical challenges (Musiał-Wysocka et al., 2019). Harvesting autologous MSCs from sources such as bone marrow or cartilage requires painful and/or invasive procedures, while the use of non-autologous cells raises concerns of immunogenicity and tumorigenesis (Hall and Watt, 1989; Charron, 2013). Additionally, culturing, expanding, transporting, and storing live MSCs requires a large investment of time, resources, and expertise (Asghar et al., 2014). These obstacles, alongside the high regulatory burden associated with the clinical use of MSCs, have greatly limited their widespread use and caused clinicians to seek more practical and readily available orthobiologics.

## 2 Materials and methods

### 2.1 Cell sources and culture

Bone marrow-derived MSCs were isolated from 5 week old PHaM mitoDendra2 (Jax stock 018385) mice, as previously described (Huang et al., 2015). Chondrocytes were isolated from 5 day old UBC mCherry mice (Jax stock 017614), as previously reported (Gosset et al., 2008). Cells were cryopreserved in liquid nitrogen then thawed and cultured for use as needed. Protocols were approved by the Institutional Animal Care and Use Committee at Cornell University. Murine cells expressing endogenous fluorophores were used for characterizing the size of the whole EV population using dynamic light scattering (DLS), and for assessing mitoEV uptake by chondrocytes with confocal imaging and flow cytometry. Human bone marrow-derived MSCs were purchased at passage two (Millipore Sigma: SCC034) and used for nanoparticle tracking, western blot, and identification of mitoEVs with flow cytometry. For all experiments using human MSCs, cells were passaged no more than 3 times, cultured to 85% confluence, rinsed thrice with PBS and serum starved for 24 h before EV isolation.

### 2.2 Extracellular vesicle isolation

For all experiments, cell-conditioned media was removed and placed on ice immediately following 24-h serum starvation. To characterize the size range of the entire MSC-derived EV population using (DLS), murine MSC cell-conditioned media (CCM) was centrifuged at 3,000 x g for 15 min. The supernatant was then processed using the Exoquick-TC ULTRA kit (System Bio: EQULTRA-20TC-1), according to manufacturer’s instructions. For nanoparticle tracking, western blot, flow cytometry, and confocal imaging studies, a validated filtration-ultracentrifugation technique was used to isolate EV fractions of specific size ranges, as previously described (Wang et al., 2021). These fractions consisted of vesicle free media, (VFM, no EVs) exosomes, (EX, 30–100 nm) and microvesicles (MVs, 100–1000 nm). Briefly, following CCM collection, adhered MSCs were lysed and placed on ice. The CCM was clarified of cellular debris with two consecutive centrifugation steps (1,000 x g for 5 min). To isolate MVs, CCM was vacuum filtered through a Steriflip filter (0.22 µm; Millipore: SE1M179M6). MVs were collected on top of the filter and were immediately placed on ice or lysed for protein analysis. The filtrate was subjected to 100,000 x g centrifugation at 4°C for 4 h in a Beckman Coulter Optima XE Ultracentrifuge. The supernatant (vesicle-free media; VFM) was removed and concentrated using an Amicon centrifugal concentrator with a 10kDa pore size. The pellet (EX fraction) was lysed and immediately placed on ice. Total whole cell lysate (WCL), MV, EX, and VFM protein content was quantified by Bradford Assay. Validation of EV isolation was performed by western blot for EV-specific markers and calcein staining/flow cytometry (Gray et al., 2015).

### 2.3 EV size characterization

The relative size distribution of EVs from MT stressed and MT protected cells was characterized by DLS (Malvern Nano ZS Zetasizer). Murine MSCs were passaged 2–3 times, cultured to 85% confluence in standard growth media at 21% O_2_ and 5% CO_2_; then rinsed thrice with phosphate buffered saline (PBS) and stimulated with the mitochondrial inhibitor rotenone/antimycin (Sigma: R8875, 0.5 μM/Sigma: A8674, 0.5 μM), treated with the mitoprotective peptide SS-31 (1 uM), or left untreated during a 24-h serum starvation prior to EV isolation. Three measurements were obtained per sample at 25°C, a detection angle of 173°, and a refractive index of 1.334 (PBS), then averaged. The experiment was repeated twice, for an *n* = 3. Percent area under the curve, maximum intensity, and mean particle size were calculated for major peaks. To obtain a more accurate quantification of sub-micron sized EVs, human MSCs were cultured as described above, then isolated using filtration/ultracentrifugation. Nanoparticle tracking was performed using a Malvern NanoSight NS300 configured with a 488 nm laser and 565 nm long pass filter for fluorescent detection. Five, 60 s videos were taken, individual particles were tracked, and hydrodynamic size was calculated via the Stokes-Einstein equation. To validate our isolation technique EX, MV, vesicle-free media, and PBS (control) were analyzed to confirm that each fraction fell within the expected size range based on previous work (Zhang et al., 2020). Samples were diluted in double filtered (.22 um) PBS to a concentration between 10^7^–10^9^ per mL and analyzed at room temperature. Size distribution of EVs in each fraction was measured using the 488 nm laser. The size distribution of mitoEVs specifically was determined by staining microvesicle fractions with the mitochondrial probe Mitotracker Red (100 nM; Thermofisher: M22425) and using the 565 nm, long pass filter. Unstained and vesicle-free samples served as negative controls.

### 2.4 Characterization of MSC secretome fractions by western blot

A total of 25 µg protein from human MSC-EV fractions isolated by filtration/ultracentrifugation was resolved by SDS-PAGE under reducing conditions (4–20%; Invitrogen Novex Wedge Well, 1.0 mM; Tris-Glycine, Mini Protein Gel, 10-well; XCell SureLock Mini). The gel was transferred to a polyvinylidene difluoride membrane (0.45 µm; 10 × 10 cm, Thermofisher), which was blocked in tris-buffered saline with 5% bovine serum albumin, then cut based on molecular weight. Sections were incubated with respective primary antibodies overnight: HSP90 (Cell Signaling: 4877S) was used as a positive loading control; Flotillin (Cell Signaling: 3436S) was used as a positive EV marker expected to be detected in all lysates (WCL, MV, EX); IκBα (Cell Signaling: 4814S) was used as cellular marker to screen for cellular contaminants in conditioned media fractions; ATP5A1 (Thermofisher:459240) and COXIV (Cell Signaling: 4850S) were used to probe for mitochondrial proteins. The membrane sections were rinsed three times with tris-buffered saline and incubated with horseradish peroxidase-conjugated secondary antibody for 1 h at room temperature with gentle rocking. All primary and secondary antibodies were used at 1:1,000 dilutions. Proteins were detected using enhanced chemiluminescence reagents and a chemiluminescent imaging device (Bio-Rad Chemidoc XRS).

### 2.5 MitoEV identification and size characterization by flow cytometry

To investigate the presence of mitoEVs, human MSC-MVs were cultured and isolated as described above, then stained with Calcein AM (10 uM; Thermofisher: C3099) to identify intact vesicles (Gray et al., 2015), and MitoTracker Deep Red (100 nM; Thermofisher: M22426) to label intact mitochondria. MVs were resuspended in double filtered PBS and immediately analyzed by flow cytometry (FACSAria Fusion, BD Biosciences). Unstained controls and EV fractions stained with individual fluorophores were prepared for each trial and used to set positive fluorescence thresholds within each experiment to account for variations in fluorescent intensitiy and instrument settings between experiments. Events positive for both Calcein and MitoTracker were considered mitoEVs. Events positive for Calcein only were considered EVs lacking whole mitochondria cargoes. Events positive for Mitotracker alone were considered free mitochondria (i.e., not contained within EVs). Data analysis was conducted using FlowJo (version 10.8; FlowJo LLC) software. To investigate the relative size distribution of mitoEVs within the isolated MV population, the backgating tool in FlowJo was used. MitoEV events were plotted based on vesicle size (FSC-H vs. FSC-A). Gates were created to encompass progressively larger size percentiles (1^st^, 50^th^, 80^th^, 90^th^, 95^th^) so that, for example, events falling within the 95^th^ percentile represented the largest 5% of all EVs. The fraction of mitoEVs within each size percentile was recorded.

### 2.6 MitoEV polarity assessment

In order to investigate the presence of functional (polarized) mitochondrial cargos, human MSC-MVs were stained with calcein AM and tetramethylrhodamine, methyl ester (TMRM; 200nM; Thermofisher: 2,668), which fluoresces red-orange only when taken up across a polarized mitochondrial membrane. Single color and unstained controls were used to set thresholds for each fluorophore, as above. Events double positive for calcein and TMRM were considered mitoEVs containing functional mitochondria.

### 2.7 MitoEV uptake by articular chondrocytes

#### 2.7.1 Flow cytometry of chondrocytes cultured with MSC-MVs

To gather quantitative data on rates of mitoEV-mediated mitochondrial transfer, PHaM mitoDendra2 MSCs were cultured to 85% confluence, rinsed thrice with PBS, and serum starved for 12 h. MSC- microvesicles were isolated via filtration as described above. Murine articular chondrocytes were harvested from 5 to 6 day old pups, as previously described (Gosset et al., 2008). Passage two to three chondrocytes were cultured to 80% confluence and incubated for 12 h in serum free media with or without murine MSC-derived MVs. Cells were then fixed and analyzed using an Attune NxT cytometer. Single cells were gated for using forward and side scatter. A negative control fluorescent threshold for dendra2 was set using non-MV treated chondrocytes. Chondrocytes fluorescing above dendra2 threshold were considered to have taken up mitoEVs.

#### 2.7.2 Confocal imaging of chondrocytes cultured with MSC-MVs

PHaM mitoDendra2 MSCs were cultured, rinsed thrice with PBS and serum starved for 24 h. Murine chondrocytes were cultured to 80% confluence on a chamber slide system (Lab-Tek: 177429), then stained with Mitotracker Red (100 nM; Thermofisher: M7512) and incubated with MSC-derived MVs for 12 h under serum starvation. Chondrocytes were then fixed on the chambered slide with 4% PFA, coverslipped with fluoromount containing DAPI nuclear stain. Chondrocyte cultures were imaged on a Zeiss LSM880 inverted i880 multiphoton microscope with a ×63 oil immersion objective using sequential, 3-channel scans; 359/457 nm (DAPI), 490/507 nm (Dendra2) 581/644 nm (Mitotracker red) excitation/emission, respectively. Z stacks were performed (14–20 slices at a step size of .38 μm) on a subset of co-fluorescent cells to investigate if MSC-derived fluorescent mitochondria were localized to the MT networks within recipient chondrocytes.

### 2.8 Statistical analysis

For DLS parameters (percent area under the curve, maximum intensity, and mean particle size), a one-way ANOVA was used, followed by Dunnett’s multiple comparison’s test to assess differences between groups (NS, RA, SS31) at each peak. To validate mitoEV quantification and mitochondrial polarity data, one-way ANOVA followed by Dunnett’s multiple comparisons tests were used to assess differences between the experimental groups (MitoTracker + calcein and TMRM + calcein, respectively), single color controls (MitoTracker, calcein, and MitoTracker, TMRM, respectively), and unstained controls. Polarity data was log transformed for statistical analysis. To assess differences between MV-treated (+MV) and non-treated (-MV) chondrocytes, data was log transformed and an unpaired t-test was used to compare groups. All statistical analyses were performed using Graphpad Prism version 8.3, with significance set at *p* ≤ 0.05.

## 3 Results

### 3.1 MSC-EV isolation validation and basic characterization

**FIGURE 1:**
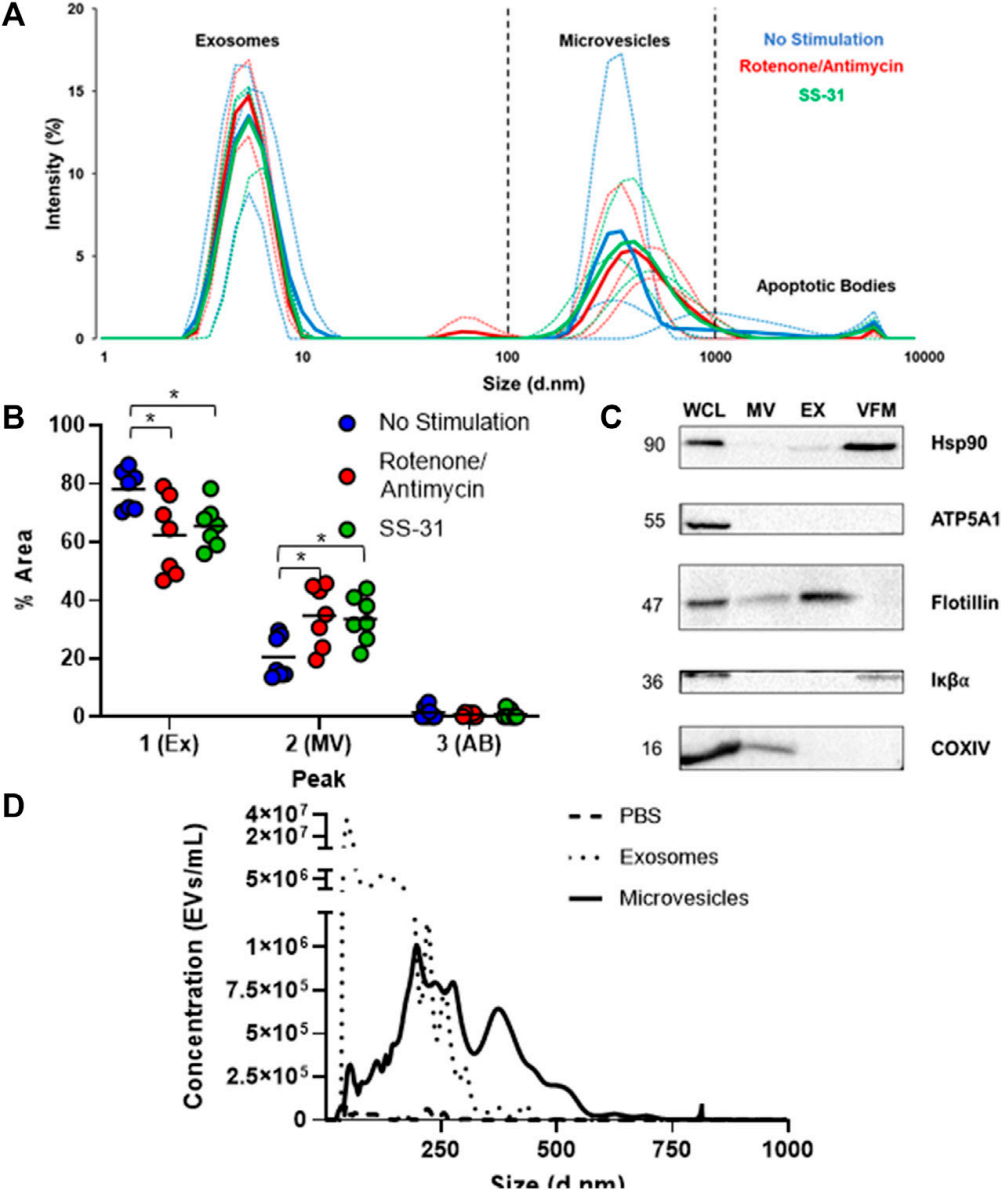
Basic characterization of human MSC-derived extracellular vesicles. **(A)** Dynamic Light Scattering (DLS) of cell-conditioned media (CCM) from murine MSCs revealed three sub-populations of EVs based on size: small, (∼5–10 nm) medium, (∼100–1000 nm) and large (5,000–10,000 nm), which likely represent exosomes, microvesicles, and apoptotic bodies, respectively. Thin lines represent individual trials (*n* = 3), thick lines represent averaged curves. **(B)** Analysis by one way ANOVA of average percent area under the curve on DLS data revealed a significantly smaller 1^st^ (exosome) peak and a significantly larger 2^nd^ (microvesicle) peak for both stimulated groups (* = *p*≤0.05). **(C)** Immunoblotting of whole cell lysate (WCL) and microvesicle (MV), exosome (EX), and vesicle free media (VFM) fractions of CCM. Ubiquitous marker HSP90 is present in WCL, EX, and VFM fractions. The EV marker flotillin is present in all fractions except VFM, and the cellular marker IκBα is absent from exosome (EX) and microvesicle (MV) fractions, as expected. MT protein ATP5A1 was found only in WCL, but COXIV (MT) was found in WCL and MVs. **(D)** Nanoparticle tracking analysis (NTA) of PBS (negative control), EX, and MV fractions indicates that EXs outnumber MVs by several orders of magnitude amongst particles less than 1μm, the maximum detection size of NTA.

To characterize the entire MSC-EV population, a size distribution was obtained by DLS. Similar to previous reports (Zhang et al., 2020), EVs clustered into three size ranges: small vesicles (5–10 nm in diameter), medium (100–1,000 nm), and a subgroup of much larger vesicles (5000–10000 nm) (Figure 1A) likely representing exosomes, microvesicles (MVs), and apoptotic bodes, respectively. The non-stimulated group had a significantly higher percent area in the first size peak than either the rotenone/antimycin or the SS-31 stimulated group. Both stimulated groups showed a significantly higher percent area in the second (medium) peak than the non-stimulated group (*p* < 0.05). Since DLS is not well suited to quantifying heterogenous populations of particles, we also performed nanoparticle tracking analysis (NTA) of EV fractions. NTA data validated our isolation protocol; particles clustered in size ranges consistent with exosomes (10–150 nm) and MVs (100–1,000 nm). The negative control diluent (double filtered PBS) was negative for EV-sized particles (Figure 1B). Next, we used Western Blot to qualitatively characterize our separated MV, EX, and vesicle free media (VFM) fractions. To confirm that the fractions were devoid of cells and cellular debris, we probed for the cellular marker, IκBα, which was detected in our whole cell lysate (WCL) but not MV or EX. The ubiquitous marker for cells and conditioned media, HSP90, was detected in WCL, EX, and VFM, as expected. Flotillin, an EV marker, was detected in WCL, MV, and EX but not VFM. Mitochondrial proteins were detected in WCL (ATP5A1, COXIV) and MV (COXIV) (Figure 1C), indicating the presence of mitochondrial cargoes in the microvesicle fraction of the MSC secretome.

### 3.2 Characterization of mitoEVs

**FIGURE 2:**
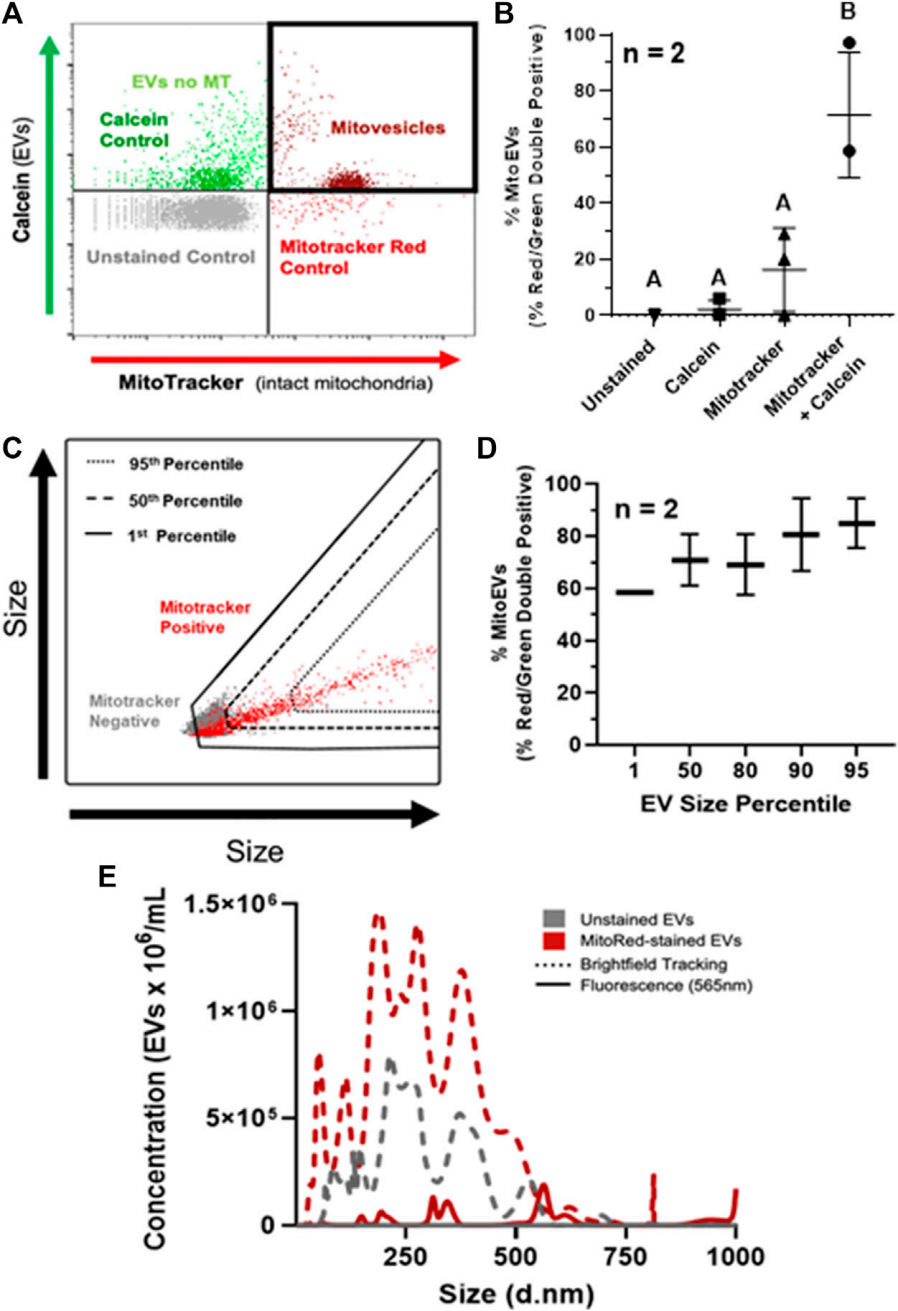
MSC-derived EVs contain mitochondria (MT). **(A)** Events fluorescing above control thresholds for Calcein AM (Green) and Mitotracker (Red) on flow cytometry were classified as EVs containing intact MT (i.e., mitoEVs; dark red). **(B)** Quantification of mitoEVs (red-green double positive events) and three control groups; Mitotracker only (▴), calcein only (■), and unstained control (▾), compared to EVs stained with calcein + Mitotracker (●). **(C)** Representative flow plot depicting backgating strategy for assessing relative sizes of mitoEVs (red) and MT-negative EVs (gray). **(D)** Backgating revealed that EVs of all sizes contain MT, but EVs lacking MT are generally small, and the majority of the largest EVs contain MT. **(E)** Fluorescent NTA revealed mitoEVs (solid red line) trend larger than the general EV population (dotted lines) and cluster into distinct sub-groups. Groups that do not share letters (Panel B) are significantly different (*p* ≤0.05) by one way ANOVA.

Quantification of mitoEVs was performed on flow cytometry. Events exceeding the fluorescence thresholds for both MitoTracker Deep Red and calcein (i.e. red-green double-positive) were considered mitoEVs (Figure 2A). An average of 71% (range 58.6–97%) of intact EVs contained MT (Figure 2B). The double stained group contained ∼7-fold higher percentage of double positive events compared to controls, indicating that the mitoEV signal was not a result of autofluorescence or artifact (Figure 2B). Backgating analysis revealed that mitoEVs were present throughout the entire size distribution of particles, however EVs lacking MT tended to be below the 50^th^ percentile for size (Figure 2C). Further, the vast majority (∼90%) of the largest EVs (95^th^ size percentile) contained MT. (Figure 2D). Because size data gathered from backgating analysis is qualitative rather than quantitative, we also performed fluorescent nanoparticle tracking analysis on MVs stained with and without MitoTracker Deep Red (Figure 2E). Fluorescent nanoparticle tracking supported our flow cytometry data; mitoEVs were found to be distributed throughout the EV size spectrum but tended to be larger than the general population. Further, this more sensitive analysis revealed that mitoEVs clustered around 3–400 nm, 600 nm, and 1 μm, suggesting the presence of distinct sub-populations of mitoEVs (Figure 2E). Unstained samples were undetectable when analyzed using the 565 nm fluorescent laser, and groups stained with MitoTracker, but analyzed with brightfield tracking did not show a shift in size distribution, validating our method for detecting mitoEVs (Figure 2E).

### 3.3 A subset of mitoEVs contain functional mitochondria

**FIGURE 3:**
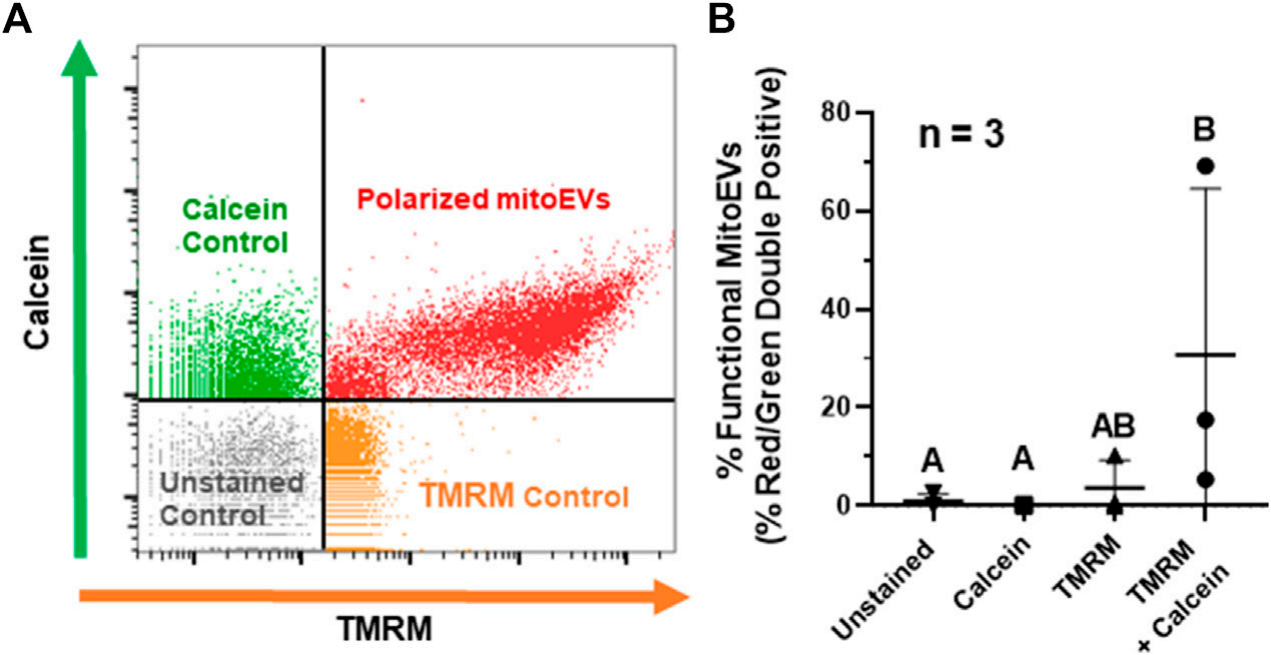
A subset of MitoEVs contain functional mitochondria. **(A)** Representative flow plot identifying polarized mitoEVs; events fluorescing above control thresholds for calcein AM (green) and TMRM (orange) were classified as mitoEVs containing functional MT (red). **(B)**. Quantification of functional mitoEVs (red-green double positive events) and three control groups; TMRM only (▴), calcein only (■), and unstained control (▾), compared to EVs stained with calcein + TMRM (●). Data is expressed as the fraction of double-positive events in each group. of percent red/green double positive showed statistically significant differences between the double stained experimental group and single color/unstained controls Groups that do not share a letter are significantly different (*n* = 3, *p* ≤0.05) by one-way ANOVA.

To characterize the functionality of mitochondrial cargoes within MSC-derived mitoEVs, we analyzed MT polarity via TMRM staining on flow cytometry, with events staining above threshold for calcein and TMR considered mitoEVs containing polarized MT (Figure 3A). An average of 31% (range 5.38–69.16%) of EVs contained functional MT, suggesting that MT polarity in mitoEVs is highly variable (Figure 3B). The double-stained experimental group contained significantly more double-positive events than unstained and calcein control groups. Although the experimental group contained a mean of 6-fold more double-positive events than the TMRM control, the difference did not reach statistical significance (*p* = 0.0735) due to high variability in the experimental group between trials (*n* = 3).

### 3.4 MitoEVs are taken up by chondrocytes and incorporated into chondrocyte mitochondrial networks

**FIGURE 4:**
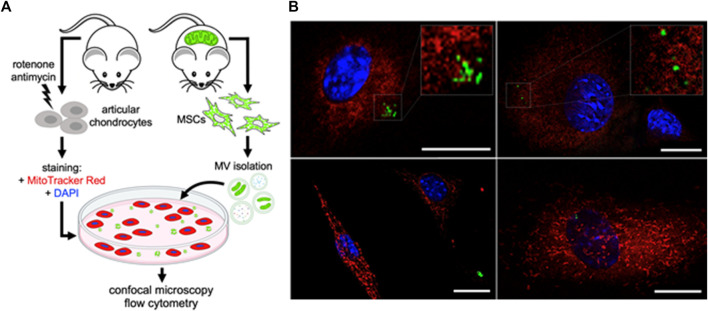
Murine Chondrocytes take up MT from MSC-derived Microvesicles **(A)** Schematic depicting experimental method for imaging non-contact intercellular MT transfer **(B)** Confocal images of MSC-MitoEVs and mitoEV mediated MT transfer. MT Transfer demonstrated with mitoEVs (green) incorporated into chondrocyte MT networks (red). Chondrocytes were co-cultured with MSC-microvesicles for 12 h, then fixed and imaged.

**FIGURE 5:**
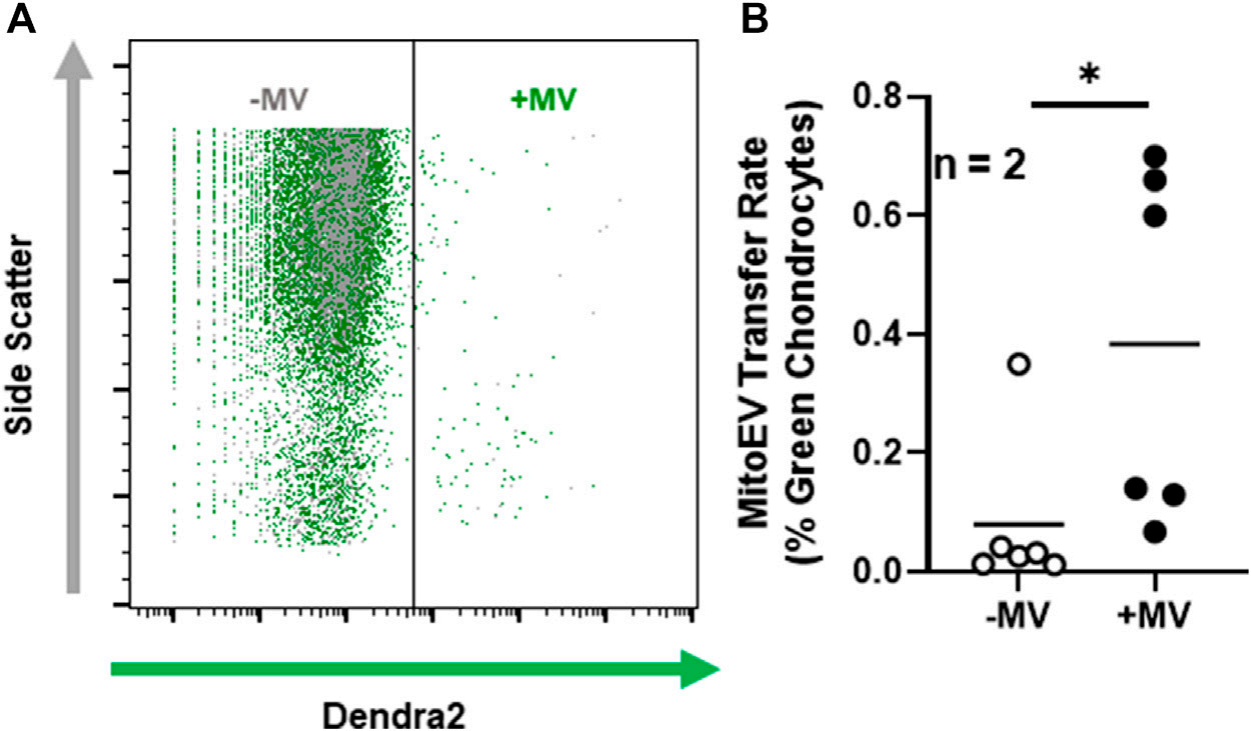
Chondrocytes take up MSC-derived MT following incubation with MitoEVs **(A)** Representative flow cytometry plots of chondrocytes incubated with (+MV) and without (-MV) microvesicles isolated from MSCs expressing endogenous, MT-specific dendra2 fluorescence. Dendra2 (green) threshold was set using control chondrocytes. Events fluorescing above threshold were considered chondrocytes having taken up mitoEVs. **(B)** Quantification of mitoEV uptake by chondrocytes in culture (*n* = 2) reveals the +MV group had a higher rate of transfer events than -MV. Data are expressed as the fraction of chondrocytes above threshold for green fluorescence. * = *p*≤0.05, by one-way ANOVA.

To interrogate whether articular chondrocytes are capable of taking up MSC-derived mitoEVs from the extracellular environment, we stained murine chondrocytes with MitoTracker Red to label the MT networks, tthen incubated them with MVs isolated from MSCs expressing endogenous, MT-specific green fluorescence (Figure 4A). Confocal imaging revealed mitoEVs located in both the extracellular space and within chondrocytes (Figure 4B). Z-stacked imaging confirmed that MSC-derived MT localized to the MT networks within chondrocytes (see Supplemental Materials for representative 3D reconstructions). To confirm the phenomenon could be replicated, imaging experiments were performed twice on two separate days. As an orthogonal approach, we also cultured unstained chondrocytes with MSC-derived MVs, and performed flow cytometry (Figure 5A). This method provided quantitative data on the rate of mitoEV-mediated MT transfer; an average of 0.4% of chondrocytes took up mitoEVs in culture (Figure 5B). Although the rate of mitoEV uptake was low and variable in our experimental system, there was a significant difference between MV-treated and untreated groups (Figure 5B, *p* < 0.05).

## 4 Discussion

This study demonstrated that human MSCs release intact, functional MT in EVs. To the best of our knowledge, this work represents the first evidence of MSC to chondrocyte, EV-mediated MT transfer. Further, we confirmed that MSC-derived mitoEVs can serve as a vehicle for MT transfer in the absence of direct cell-cell interactions between MSCs and chondrocytes. Our findings are consistent with previous reports, where Phinney et al. found that MSCs undergoing oxidative stress produced vesicles containing dysfunctional, partially depolarized MT to outsource mitophagy. Macrophages engulfed these vesicles and repurposed functional components, improving bioenergetics for both the MSC and macrophage (Phinney et al., 2015). Morrison et al. used mitochondrial organelle staining of MSC-derived EVs and flow cytometry to show that mitoEVs released by MSCs can be incorporated by macrophages into their MT networks. MitoEV uptake by macrophages enhanced phagocytosis by promoting oxidative phosphorylation, and improved their ability to treat lipopolysaccharide-induced lung injury *in vivo* (Morrison et al., 2017). Consistent with our findings, evidence that cells release both functional and non-functional MT in EVs suggests mitoEV transfer is a dynamic, context-driven process, likely involving crosstalk (i.e., bidirectional mitoEV transfer) between donor and recipient cells (Morris and Hollenbeck, 1993; Plotnikov et al., 2008). Studies of contact intercellular MT transfer, involving direct interactions between donor and recipient cells in other tissues indicate that calcium signaling and gap junction proteins play an important role (Islam et al., 2012; Hayakawa et al., 2016). However, the mechanisms of EV-mediated MT transfer are unclear, and further investigation is warranted. In our proof-of-concept experimental design, we found a low rate of mitoEV uptake by chondrocytes *in vitro*. However, it should be noted that several factors, including culture time and MV numbers were not optimized for these experiments. In similar work, where chondrocytes and MSCs were directly co-cultured, the frequency of MT transfer was higher than reported here for mitoEV-mediated transfer; between 1–15% depending on experimental parameters, including culture time, cell ratios, and environmental conditions (Bennett et al., 2019; Fahey et al., 2022). Therefore, we anticipate that future work to optimize experimental parameters and identify mechanisms mediating MSC-chondrocyte mitoEV transfer will allow us to manipulate the rate of mitoEV release by MSCs and uptake by chondrocytes.

To investigate functionality of MT packaged into mitoEVs we used TMRM staining, where fluorescent intensity is directly proportional to the inner MT membrane potential (Brand and Nicholls, 2011). Using this technique, we found that a subset (at least 5%, up to 70%) of mitoEVs contain functional MT, meaning that they have a competent electron transport chain and are capable of producing ATP. We suspect that the high variability in TMRM-staining between experiments is reflective of the exquisite sensitivity of mitochondria to depolarization, and subtle differences in MT stress induced by EV isolation and analysis techniques, especially flow cytometry, although further studies would be required to confirm this. Our preliminary experience suggests that while fluorescence activated cell sorting (FACS) of mitoEV subpopulations is possible, this approach is damaging to mitoEV polarity, and too time, effort, and cost-intensive to be practical at this time (Kormelink et al., 2016). However, our data suggest the possibility of more scalable, size-dependent methods of mitoEV isolation; We found that more than 70% of MSC-MVs are mitoEVs, a higher percentage than previously reported (Morrison et al., 2017). This is likely due to several experimental differences, such as cell of origin, EV isolation technique, and EV identification method. However, further size analysis of our mitoEV population indicated that nearly all of the MVs >500 nm contain mitochondria. Further studies to investigate methods of isolating mitoEVs based on a size will be necessary to investigate the functional impact of mitoEV uptake on injured chondrocytes. Based on work suggesting MSC donation of functional MT to injured cells can rescue cell viability and promote tissue healing, optimizing functionality of mitoEVs during isolation would be an important consideration for the investigation of mitoEVs as a regenerative orthobiologic therapy (Pacak et al., 2015; Berridge et al., 2016; Jiang et al., 2016; Konari et al., 2019).

Our data suggest mitoEVs themselves are not a homogenous population, but rather a diverse collection of vesicles containing distinct mitochondrial cargoes, with a range of functionality. Distinct mitoEV sub-populations may play biologically distinct roles, including cellular rescue through intercellular mitochondrial transfer, outsourcing of mitophagy and diverse signaling functions (Phinney et al., 2015; Morrison et al., 2017; Sansone et al., 2017; Zhang et al., 2020; Peruzzotti-Jametti et al., 2021). DLS size data further supports this. We found that agents known to affect mitochondrial dynamics and function shift the size profile of EV subgroups released by MSCs; When MSCs were stimulated with either a mitoprotective peptide or a MT specific stressor, we observed a significant shift towards a larger 100–1,000 nm, “medium” peak, presumably MVs. Although our current experimental methods did not allow us to determine why both groups responded similarly, these data suggest mitoEV release is responsive to changes in cellular MT function. It is possible that following rotenone/antimycin-induced MT stress, MSCs increased packaging of dysfunctional MT into MVs, consistent with the previous work demonstrating stressed MSCs release unhealthy MT into EVs to outsource mitophagy (Phinney et al., 2015). Following mitoprotective treatment with SS-31, MSCs may similarly increase export of healthy, functional MT in mitoEVs. Further research into the origin, mechanisms of release, and function of mitoEV sub-populations is necessary to elucidate how MSC MT function is related to mitoEV biogenesis.

Measurement of particles smaller than 200 nm (i.e., exosomes) was limited to size and protein analysis. Neither of the MT proteins we probed for (COXIV an outer MT membrane protein and ATP5A1, an enzyme that catalyzes synthesis of ATP) was present in the exosome fraction. This may be due to the specific proteins we chose to investigate, rather than an indicator that MSC-exosomes do not transport MT components. Previous analysis has shown that exosomes may be more likely to contain miRNA and mtDNA, as opposed to the MT membrane proteins and intact MT seen in larger vesicles such as MVs (Phinney et al., 2015; Torralba et al., 2018; D’Acunzo et al., 2021). As techniques improve and high resolution, microparticle optimized flow cytometers become more widely available, characterization of individual mitoEV sub-populations will become more straightforward.

Anunderlying goal of this study was to characterize MSC-derived mitoEVs and investigate their potential suitability as vehicles for non-contact, acellular MT delivery in therapeutic applications. The properties that make EVs effective in long range endogenous communication, also make them excellent candidates for regenerative therapy. EV lipid bilayers are durable enough to retain structural integrity after freeze-thawing and remain intact following intraarticular injection (Li et al., 2019). EVs are non-replicating and have low levels of major histocompatibility complex proteins, posing minimal risk of immunogenicity or tumorigenicity (Yeo et al., 2013; Zhu et al., 2017; Tsiapalis and O’Driscoll, 2020). We confirmed the presence of functional MT in MSC-MVs, identified evidence of distinct sub-populations of mitoEVs, and found that, although MT are present in MVs of all sizes, large MVs are much more likely to contain MT. Further, we observed evidence that stressed chondrocytes will take up MT packaged in mitoEVs in the absence of physical interactions with the parent MSCs. The methods developed in this study will allow investigation into the effects of these mitoEVs after uptake and the mechanisms underlying this phenomenon, as well as those behind why distinct sub-populations such as functional and dysfunctional mitoEVs are released.

## Data Availability

The original contributions presented in the study are included in the article/Supplementary Material, further inquiries can be directed to the corresponding author.
