# Supplementary figures and images for: Human mesenchymal stromal cells release functional mitochondria in extracellular vesicles
Source: Front Bioeng Biotechnol. 2022 Aug 19;10:870193. doi: 10.3389/fbioe.2022.870193 (PMC9446449; doi:10.3389/fbioe.2022.870193)

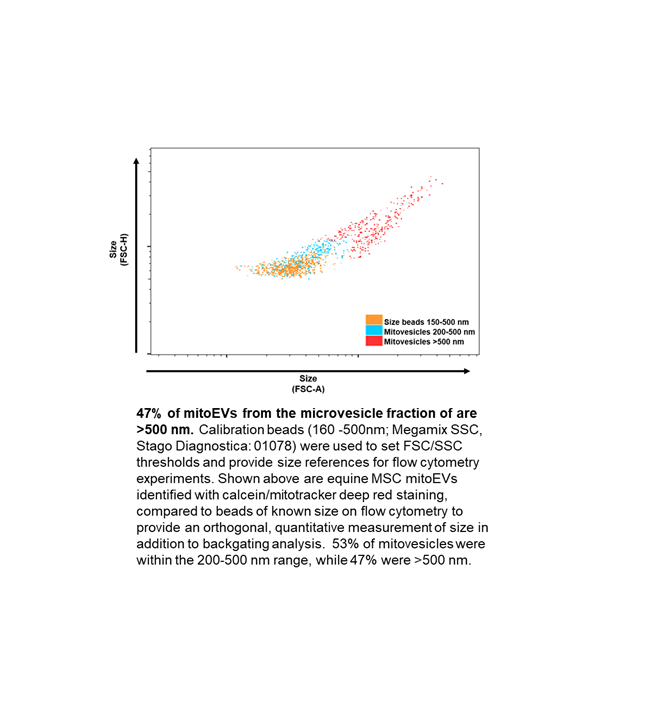

Supplement: Supplementary file 3 [file Image2.tif]

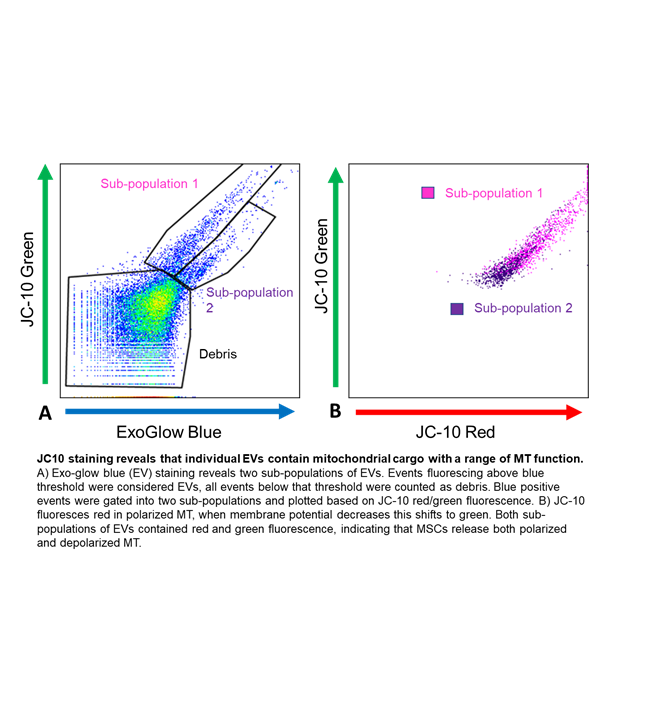

Supplement: Supplementary file 4 [file Image1.tif]
